# Multiple cancer cell types release LIF and Gal3 to hijack neural signals

**DOI:** 10.1038/s41422-024-00946-z

**Published:** 2024-03-11

**Authors:** Qun Xu, Ying Cao, Fanni Kong, Jiaqi Liu, Xin Chen, Yifei Zhao, Chin-Hui Lai, Xin Zhou, Hao Hu, Wei Fu, Jian Chen, Jing Yang

**Affiliations:** 1 State Key Laboratory of Membrane Biology, School of Life Sciences, Peking University, Beijing, China; 2 Center for Life Sciences, Academy for Advanced Interdisciplinary Studies, Peking University, Beijing, China; 3 Department of General Surgery, Peking University Third Hospital, Beijing, China; 4 Department of Urology, Peking University People’s Hospital, Beijing, China; 5 Peking University Third Hospital Cancer Center, Beijing, China; 6 Chinese Institute for Brain Research, Beijing, China; 7 IDG/McGovern Institute for Brain Research, Peking University, Beijing, China

**Keywords:** Cancer, Immunology

## Abstract

Neural signals can significantly influence cancer prognosis. However, how cancer cells may proactively modulate the nervous system to benefit their own survival is incompletely understood. In this study, we report an overlapping pattern of brain responses, including that in the paraventricular nucleus of the hypothalamus, in multiple mouse models of peripheral cancers. A multi-omic screening then identifies leukemia inhibitory factor (LIF) and galectin-3 (Gal3) as the key cytokines released by these cancer cell types to trigger brain activation. Importantly, increased plasma levels of these two cytokines are observed in patients with different cancers. We further demonstrate that pharmacologic or genetic blockage of cancer cell-derived LIF or Gal3 signaling abolishes the brain responses and strongly inhibits tumor growth. In addition, ablation of peripheral sympathetic actions can similarly restore antitumor immunity. These results have elucidated a novel, shared mechanism of multiple cancer cell types hijacking the nervous system to promote tumor progression.

## Introduction

It has become increasingly recognized that the nervous system can significantly influence cancer prognosis. Indeed, the emerging frontier of cancer neuroscience has garnered research attention in the past years.^1–3^ For instance, the presence of sympathetic or parasympathetic innervations in prostate tumors correlated with poorer survival of patients.^4^ Conversely, the incidence of prostate cancer decreased in the male population inflicted by spinal cord injuries that conceivably abrogated the efferent sympathetic or parasympathetic actions.^5^ In the women taking β-adrenergic receptor-antagonizing drugs (i.e., beta blockers) that chronically attenuated sympathetic signaling, the rates of metastasis or mortality became significantly lower for breast cancer^6,7^ or ovary cancer.^8^ In addition, the use of beta-blockers improved the survival outcomes of non-small-cell lung cancer patients receiving radiotherapy,^9^ chemotherapy,^10^ or immunotherapy.^11^ Therefore, the comprehensive knowledge of cancer neuroscience holds the promise of revealing novel diagnostic or therapeutic strategies against those dreadful human diseases.

Clinical observations on the involvement of the body’s nervous system in cancers can be recapitulated in mouse models, which offer valuable mechanistic insights. For example, the local ablation of sympathetic or parasympathetic innervations in the mouse prostate elucidated their disparate roles in tumorigenesis or tumor progression.^4^ Surgical or pharmacological blockage of neural innervations in the mouse stomach suppressed the Wnt-mediated cell proliferation, thus ameliorating the onset and growth of gastric cancer.^12^ In addition, the beta-blocker treatment in mice slowed the metastasis of breast cancer by decreasing the infiltration of tumor-associated macrophages.^13^ Furthermore, inhibiting the neurotransmitter glutamate signaling via the *N*-methyl-*D*-aspartate receptor reduced the invasiveness of the mouse pancreatic neuroendocrine tumor or pancreatic ductal adenocarcinoma.^14^

The complexity of cancer–neural interactions calls for more in-depth investigations. Notably, recent studies have begun suggesting that cancer cells proactively engage the nervous system to benefit their survival. For instance, cancer cells in a mouse model of pancreatic ductal adenocarcinoma could release nerve growth factor (NGF), promoting the infiltration of sympathetic axons to exaggerate tumor progression.^15^ Similarly, various human cell lines of prostate cancer, colon cancer, or pancreatic cancer produced the precursor of brain-derived neurotrophic factor (pro-BDNF), which led to the increase of neural innervations to tumors.^16^ Moreover, in the mouse allograft models of MC38 colon adenocarcinoma and Pan02 pancreatic ductal adenocarcinoma or the *Apc*^*min/+*^ model of intestinal tumors, peripheral cancers could trigger the activation of catecholaminergic neurons in the ventrolateral medulla, facilitating tumor growth via an inhibition of CD8^+^ T cells.^17^ In addition, the pituitary production of α-melanocyte-stimulating hormone (i.e., α-MSH) occurred in the mouse allograft models of Lewis lung carcinoma (LLC), MC38 colon adenocarcinoma, MCA205 fibrosarcoma, or B16-F10 melanoma, stimulating the generation of myeloid-derived suppressor cells (MDSCs) to dampen antitumor immunity.^18^ Despite these research advances, pathophysiological mechanisms underlying cancer–neural crosstalk remain incompletely charted. In particular, how cancer cells may release specific signaling molecules to induce brain responses needs to be clarified.

## Results

### An overlapping pattern of brain responses to multiple types of peripheral cancers

**Fig. 1:**
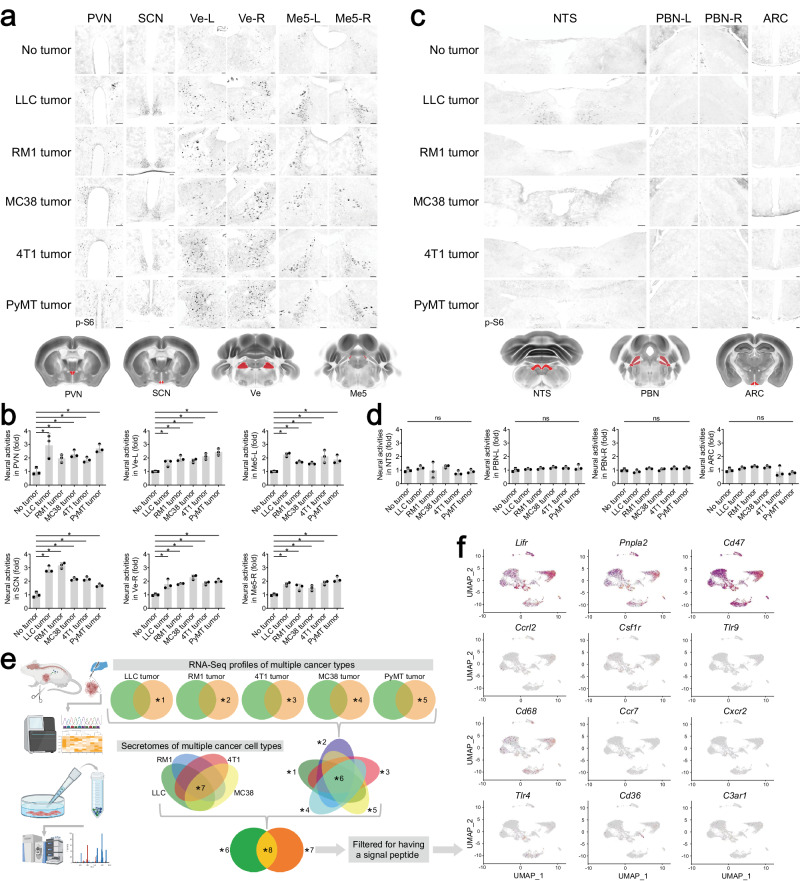
An overlapping pattern of brain responses in multiple mouse models of peripheral cancers. **a**–**d** Mouse allograft models of LLC, RM1, MC38, or 4T1 cells and the *MMTV-PyMT* mouse model were utilized. Brain responses were assessed by the p-S6 immunostaining. Representative images of the PVN, SCN, Ve-L/-R, and Me5-L/-R (**a**) or the NTS, PBN-L/-R, and ARC (**c**) were shown. Scale bars, 100 μm. Neural activities in the indicated brain regions were quantified (**b**, **d**). Data are shown as mean ± SD. One-way ANOVA test; ns, not significant; *P < 0.05. **e**, **f** A multi-omic screening for the cancer cell-derived factor(s) triggering brain responses. The scheme of integrating RNA-seq data of different cancer types and secretomic analyses of cultured cancer cells was illustrated (**e**). The published scRNA-seq dataset of mouse trigeminal ganglionic neurons (GSE213105) was exploited to identify candidate proteins for functional examination (**f**).

We sought to explore the mechanism of peripheral cancers communicating with the central nervous system. As the entry point of the study, several mouse allograft models were utilized, i.e., LLC lung cancer, RM1 prostate cancer, MC38 colon cancer, and 4T1 breast cancer. Notably, these allograft models have been commonly exploited in cancer immunology to investigate antitumor immunity and MDSCs in immune-competent wild-type mice.^19,20^ Mice were subcutaneously implanted with each cancer cell line, and brain tissues of tumor-bearing mice were then processed for the immunostaining of phospho-ribosomal protein S6 (p-S6), a specific marker for neuronal activation.^21^ We comprehensively assessed brain regions ranging rostrocaudally from olfactory bulbs to the brainstem. Surprisingly, we identified a shared pattern of brain responses under all the tumor-bearing conditions examined, including that in the paraventricular nucleus of the hypothalamus (PVN), a central brain region initiating efferent sympathetic signals (Fig. 1a, b). Further, responses of the suprachiasmatic nucleus (SCN), the left and right vestibular nucleus (Ve-L/-R), the left and right mesencephalic nucleus of the trigeminal nerve (Me5-L/-R), the red nucleus (RN), the hypoglossal nucleus (12N), and the left and right motor nucleus of the trigeminal nerve (5N-L/-R) were triggered in these allograft models (Fig. 1a, b; Supplementary information, Fig. S1a, b). On the other hand, there were no detectable neural activities in the brain regions related to visceral or metabolic signaling, e.g., the nucleus of the solitary tract (NTS), the left and right parabrachial nucleus (PBN-L/-R), and the arcuate nucleus (ARC) (Fig. 1c, d), exemplifying the specificity of cancer-induced brain activation. In parallel, we looked into the orthotopic allograft lung cancer model of LLC cells^22^ or the orthotopic prostate cancer model of RM1 cells.^23^ These two models of orthotopic tumors exhibited brain responses comparable to those in the heterotopic subcutaneous tumors (Supplementary information, Fig. S2a, b).

In addition to the allograft cancer models, we assessed the brain activation of *MMTV-PyMT* mouse, a standard genetic model of orthotopic breast cancer.^24^ Importantly, this genetic model exhibited the same set of responsive and non-responsive brain regions as the above allograft models (Fig. 1a–d; Supplementary information, Fig. S1a, b). These results suggested that despite their differential origins and mutational spectra, multiple types of peripheral cancers could induce an overlapping pattern of brain responses.

To determine whether the physical burden of a tumor might underlie the commonality of brain responses to different cancers, we implanted the wild-type mice with a pseudo-tumor made of medical-grade silicon. Although these pseudo-tumors effectively mimicked the volume and weight of “real” tumors, they failed to cause any neural activities in the signature brain regions, including the PVN, SCN, Ve-L/-R, and Me5-L/-R (Supplementary information, Fig. S3a, b). This observation mostly ruled out the involvement of physical properties of peripheral tumors in inducing brain activation.

### Leukemia inhibitory factor (LIF) and galectin-3 (Gal3) as the cancer cell-derived factors triggering brain responses

We considered the possibility that multiple cancer types might release the same factor(s) to trigger the overlapping pattern of brain activation. To pursue this hypothesis, we profiled the transcriptomes of tumors from LLC, RM1, MC38, and 4T1 allograft models and *MMTV-PyMT* genetic model by RNA sequencing (RNA-seq) (Fig. 1e), and identified ~2000 candidate genes exhibiting a shared expression pattern among different cancer types. In parallel, proteins in the culture media of LLC, RM1, MC38, or 4T1 cells were examined by proteomic analyses (Fig. 1e). We found that 114 proteins were commonly present in the secretomes of these cancer cell lines, 89 of which were included in the list of candidate genes identified by RNA-seq profiling. Among them, 61 candidate proteins were then filtered for being a bona fide secreted factor, i.e., possessing a signal peptide, 11 of which have the specific receptor(s) documented (Supplementary information, Table S1). We further analyzed the expression of these receptors in the published single-cell RNA-seq (scRNA-seq) dataset of mouse trigeminal ganglionic neurons (GSE213105),^25^ whose axonal projections control Me5-L/-R and 5N-L/-R, the signature regions activated by multiple cancer types. Through this multi-omic screening, three candidate proteins (LIF, pigment epithelium-derived factor, and thrombospondin-1) were obtained for in-depth functional examination (Fig. 1f).

**Fig. 2:**
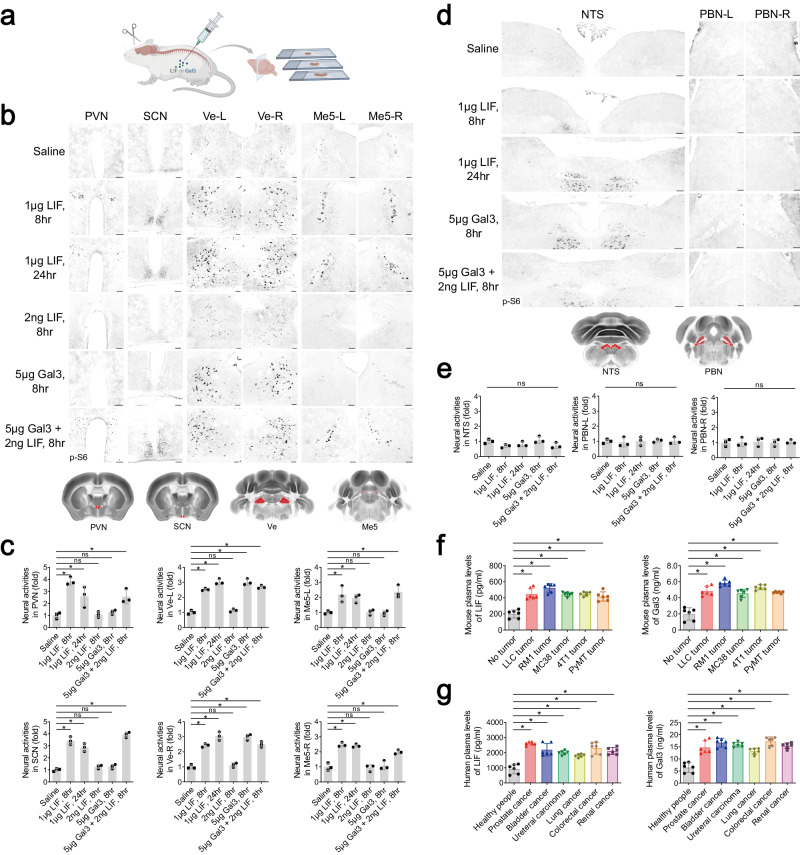
LIF and Gal3 are the cancer cell-derived factors that cooperatively activate specific brain regions. **a**–**e** Non-tumor-bearing C57BL/6 wild-type mice were administered with the indicated amounts of recombinant mouse LIF or Gal3 protein. Brain responses were assessed by the p-S6 immunostaining. The scheme of the experimental procedure was illustrated (**a**). Representative images of the PVN, SCN, Ve-L/-R, and Me5-L/-R (**b**) or the NTS and PBN-L/-R (**d**) were shown. Scale bars, 100 μm. Neural activities in the indicated brain regions were quantified (**c**, **e**). Data are presented as mean ± SD. One-way ANOVA test; ns not significant; *P < 0.05. **f** Mouse allograft models of LLC, RM1, MC38, or 4T1 cells and the *MMTV-PyMT* mouse model were utilized. Plasma levels of LIF and Gal3 in different mouse models were examined by enzyme-linked immunosorbent assay (ELISA). Data are presented as mean ± SD. One-way ANOVA test; *P < 0.05. **g** Plasma levels of LIF and Gal3 in human patients with different peripheral cancers were measured by ELISA. Data are presented as mean ± SD. One-way ANOVA test; *P < 0.05.

We treated the non-tumor-bearing wild-type mice with each recombinant protein of the three candidates, testing whether one could recapitulate the same pattern of brain responses to peripheral cancers (Fig. 2a). Remarkably, a single intraperitoneal injection of 1 μg mouse LIF could effectively achieve the activation of signature brain regions, i.e., PVN, SCN, Ve-L/-R, Me5-L/-R, RN, 12N, and 5N-L/-R, for up to 24 h (Fig. 2b, c; Supplementary information, Fig. S4a, c). Meanwhile, reminiscent of that observed in the tumor-bearing mice, this LIF treatment did not cause detectable neural activities in the NTS or PBN-L/-R (Fig. 2d, e). In addition, plasma levels of LIF protein were significantly upregulated in all the allograft models of LLC, RM1, MC38, or 4T1 cells, as well as in the tumor-bearing *MMTV-PyMT* mice (Fig. 2f; Supplementary information, Fig. S2c). Moreover, increased plasma levels of LIF were detected in patients with multiple cancer types, e.g., prostate cancer, bladder cancer, ureteral cancer, non-small cell lung cancer, colorectal cancer, and renal cancer (Fig. 2g). These results supported LIF as a cancer cell-derived factor that triggers brain responses.

However, it came to our attention that the intraperitoneal administration of 1 μg recombinant LIF protein resulted in plasma levels of ~50 ng/mL, which would be 100-fold higher than those in the tumor-bearing mice. On the other hand, although a single injection of 2 ng LIF led to its plasma levels comparable to those of the tumor-bearing conditions, this low dose was not sufficient to elicit brain activation (Fig. 2b, c; Supplementary information, Fig. S4b, d). This phenomenon raised the challenging issue that additional cancer cell-derived factor(s) might exist to function cooperatively with LIF in neural activation. To this end, we moved on to laboriously screen the commercially available recombinant proteins of the remaining 60 candidate secreted factors (Supplementary information, Table S1) in the hope of identifying one that could induce neural activity under the condition of the low-dose LIF treatment. We managed to find that while the intraperitoneal injection of 5 μg galectin-3 (Gal3) alone only induced neural activities in the Ve-L/-R of non-tumor-bearing mice, it acted in a cooperative manner with 2 ng LIF to trigger responses of all the signature brain regions (Fig. 2b, c; Supplementary information, Fig. S4b, d). As an aside, both recombinant LIF and Gal3 proteins showed a relatively fast clearance in the blood (t_1/2_ of LIF = 2.9 min and t_1/2_ of Gal3 = 3.2 min). Notably, plasma levels of Gal3 were significantly upregulated in the allograft models of LLC, RM1, MC38, or 4T1 cells and the *MMTV-PyMT* genetic model (Fig. 2f; Supplementary information, Fig. S2c). Further, Gal3 plasma levels markedly increased in patients with different cancers (Fig. 2g). These results together identified LIF and Gal3 as the key factors released by multiple cancer types to communicate with the brain.

### Blockage of LIF and Gal3 signaling to the brain inhibits tumor progression

**Fig. 3:**
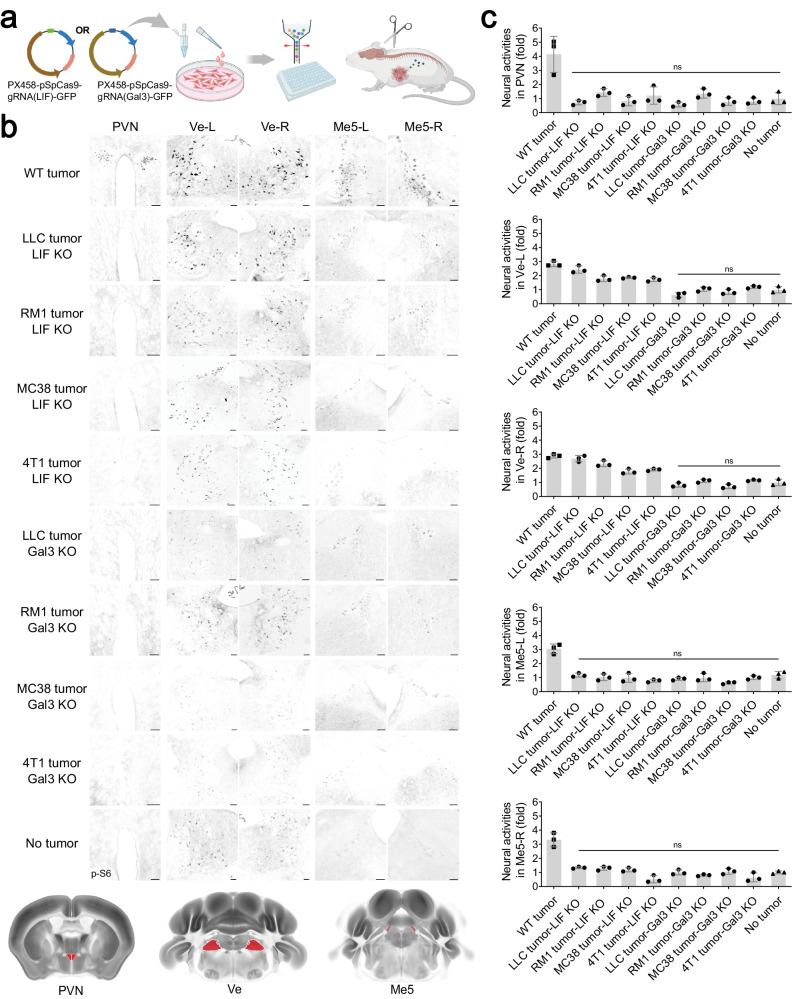
Cancer cell-derived LIF and Gal3 are essential for brain responses. **a**–**c** LLC, RM1, MC38, or 4T1 cancer cells with LIF KO or Gal3 KO were generated by CRISPR/Cas9 and then tested in mouse allograft models. Brain responses were assessed by the p-S6 immunostaining. The scheme of the experimental procedure was illustrated (**a**). Representative images of the PVN, SCN, Ve-L/-R, and Me5-L/-R were shown (**b**). Scale bars, 100 μm. Neural activities in the indicated brain regions were quantified (**c**). Data are presented as mean ± SD. One-way ANOVA test; ns, not significant; *P < 0.05.

We next explored the disease relevance of cancer cell-derived LIF and Gal3 signaling to the brain. LLC, RM1, MC38, or 4T1 cells with the genetic deletion of LIF or Gal3 were generated by CRISPR/Cas9 and then tested in the mouse allograft models (Fig. 3a; Supplementary information, Fig. S5). Significantly, LIF knockout (KO) in these cells blocked the cancer-induced activities in the signature brain regions, such as the PVN and Me5-L/-R (Fig. 3b, c). Intriguingly, LLC, RM1, MC38, or 4T1 cells with the LIF deletion did not entirely abolish neural activities in the Ve-L/-R (Fig. 3b, c), which appeared in accordance with the above observation that Gal3 alone could stimulate this brain nucleus (Fig. 2b, c). In parallel, Gal3 KO in cancer cells strongly abrogated neural activities in all the signature brain regions of tumor-bearing mice (Fig. 3b, c). These results validated the cooperative action of cancer cell-derived LIF and Gal3 in mediating brain responses to multiple types of peripheral tumors.

**Fig. 4:**
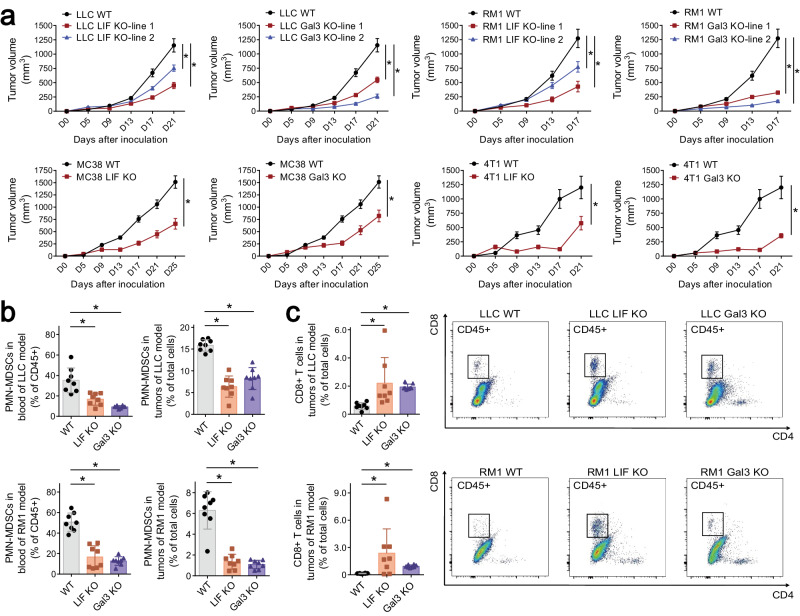
Blockage of LIF or Gal3 signaling inhibits tumor progression. **a**–**c** LLC, RM1, MC38, or 4T1 cancer cells with LIF KO or Gal3 KO were examined in mouse allograft models. Tumor growth rates under the indicated conditions were monitored (**a**). *n* = 8. Data are shown as mean ± SD. Two-way ANOVA test; *P < 0.05. MDSCs in the blood or tumors under the indicated conditions were quantified by fluorescence-activated cell sorting (FACS) (**b**). CD8^+^ T cells in the tumors under the indicated conditions were examined by FACS (**c**). Data are shown as mean ± SD. One-way ANOVA test; *P < 0.05.

Blockage of brain activation by LIF KO or Gal3 KO delayed tumor growth in the mouse allograft models of LLC, RM1, MC38, or 4T1 cells (Fig. 4a). In further support of the critical role of LIF and Gal3 signaling, we daily treated the mice bearing “wild-type” tumors with EC330, a small-molecule inhibitor of LIF.^26^ This pharmacologic approach dampened brain responses in the signature brain regions, e.g., PVN, SCN, and Me5-L/-R (Supplementary information, Fig. S6a). Meanwhile, consistent with the findings with LIF KO, neural activities in the Ve-L/-R persisted in those tumor-bearing mice treated with EC330 (Supplementary information, Fig. S6a). Of importance is that the EC330 treatment delayed tumor growth in the mouse allograft models of “wild-type” LLC, RM1, MC38, or 4T1 cells (Supplementary information, Fig. S6b). Similarly, the mice bearing “wild-type” tumors exhibited no brain response in all the signature regions when treated with the Gal3 small-molecule inhibitor GB1107 (Supplementary information, Fig. S7a).^27^ Further, this pharmacologic inhibition of the Gal3 signal led to the strong suppression of tumor growth (Supplementary information, Fig. S7b). These results elucidated the function of cancer cell-derived LIF and Gal3 signaling to the brain in modulating tumor progression.

### Sympathetic signaling promotes MDSC generation for tumor progression

Research has documented MDSCs, which can be further categorized as polymorphonuclear MDSCs (PMN-MDSCs) or monocytic MDSCs (M-MDSCs), in suppressing CD8^+^ T cell-mediated antitumor immunity.^28–30^ We found that the presence of PMN-MDSCs and M-MDSCs diminished in the blood and tumors of mouse allograft models of LIF KO or Gal3 KO cells (Fig. 4b). Accordingly, the recruitment of CD8^+^ T cells increased within the allograft tumors of LIF KO or Gal3 KO cells compared to their parental “wild-type” cells (Fig. 4c). These results revealed that blockage of cancer cell-derived LIF and Gal3 signaling to the brain could enhance antitumor immunity.

**Fig. 5:**
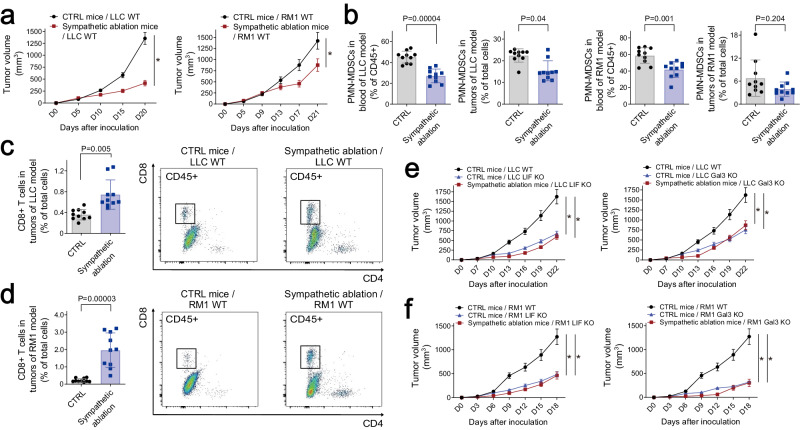
Cancer cell-derived LIF and Gal3 act via sympathetic signaling to facilitate MDSC generation. **a**–**d** *Th-Cre;TrkA*^*fl/fl*^ (sympathetic ablation) and control *Th-Cre;TrkA*^*+/+*^ littermates were utilized for LLC or RM1 allograft models. Tumor growth rates under the indicated conditions were monitored (**a**). *n* = 10. Data are presented as mean ± SD. Two-way ANOVA test; *P < 0.05. MDSCs in the blood or tumors under the indicated conditions were quantified by FACS (**b**). CD8^+^ T cells in the tumors of LLC (**c**) or RM1 (**d**) allograft models were examined by FACS. Data are presented as mean ± SD. Student’s *t*-test. **e**, **f** LLC (**e**) or RM1 (**f**) cancer cells with LIF KO or Gal3 KO were assessed in the allograft models with sympathetic ablation. Tumor growth rates under the indicated conditions were monitored. *n* = 10. Data are presented as mean ± SD. Two-way ANOVA test; *P < 0.05.

We thus sought to determine the neuroimmune mechanism afforded by such cancer-induced brain responses. MDSCs are primarily generated through myelopoiesis in lymphoid organs such as the bone marrow and the spleen. Because cancer cell-derived LIF and Gal3 cooperatively activated the PVN, a central brain region that controls efferent sympathetic action, we explored whether local sympathetic inputs in specific lymphoid organs might modulate MDSC generation. We took advantage of the *Th-Cre;TrkA*^*fl/fl*^ mouse model, in which the NGF high-affinity receptor TrkA is deleted in sympathetic neurons. Our previous works with advanced imaging techniques showed the complete ablation of sympathetic inputs in the bone marrow and the spleen of *Th-Cre;TrkA*^*fl/fl*^ mice.^31,32^ *Th-Cre;TrkA*^*fl/fl*^ (i.e., sympathetic ablation) and control *Th-Cre;TrkA*^*+/+*^ littermates were examined in mouse allograft models. We compared brain activation in these tumor-bearing mice, revealing similar levels of neural activities in the signature regions between sympathetic ablation and control conditions (Supplementary information, Fig. S8a, b). Further, there was no significant change in plasma LIF or Gal3 levels of the tumor-bearing mice with sympathetic ablation (Supplementary information, Fig. S8c). These findings substantiated that cancer-induced brain responses would act upstream of sympathetic signaling. However, tumor growth in the *Th-Cre;TrkA*^*fl/fl*^ mice was significantly inhibited (Fig. 5a), which correlated with decreased MDSCs but increased CD8^+^ T cells in the blood and tumors of these mice (Fig. 5b–d). We further tested LIF KO or Gal3 KO cancer cells in *Th-Cre;TrkA*^*fl/fl*^ and control littermates. Importantly, growth rates of LIF KO or Gal3 KO tumors were comparable between sympathetic ablation and control conditions (Fig. 5e, f), showing no additive effect of the genetic deletion of LIF or Gal3 in this context. These results supported the notion that LIF and Gal3 could act via sympathetic signaling to promote MDSC generation for tumor progression.

**Fig. 6:**
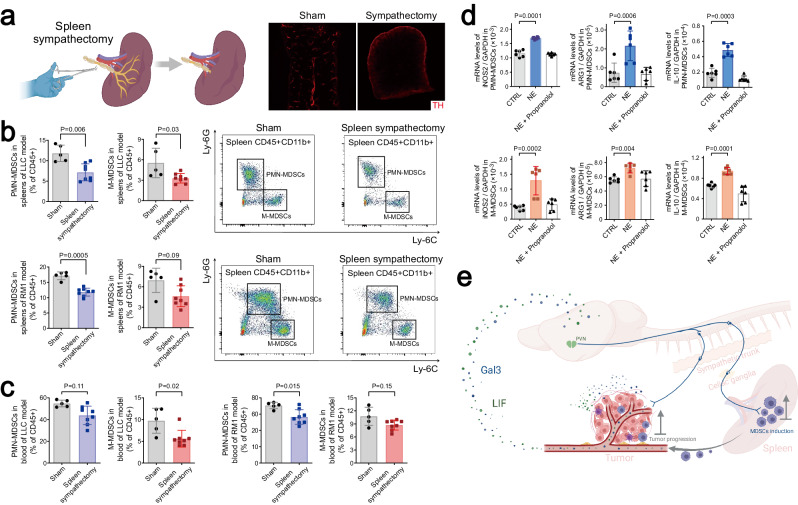
Sympathetic signaling directly promotes the immunosuppressive function of MDSCs. **a**–**c** C57BL/6 wild-type mice were subjected to spleen sympathectomy and then utilized for LLC or RM1 allograft models. Sympathetic axons in the spleen after sham surgery or sympathectomy were visualized by the immunostaining of tyrosine hydroxylase (**a**). MDSCs in the spleens (**b**) and the blood (**c**) under the indicated conditions were quantified by FACS. Data are presented as mean ± SD. Student’s *t*-test. **d** PMN-MDSCs or M-MDSCs were FACS-sorted from the spleens of tumor-bearing mice and in vitro treated with NE in combination with the β2-adrenergic receptor antagonist propranolol. mRNA levels of immunosuppressive genes were examined by qPCR analyses. Data are presented as mean ± SD. One-way ANOVA test. **e** Diagram of cancer cell-derived LIF and Gal3 hijacking the nervous system to promote tumor progression.

We next exploited the surgical approach of sympathectomy to specifically remove sympathetic inputs in the spleen of mice (Fig. 6a), which were then utilized for allograft cancer models. Reminiscent of that observed with sympathetic ablation, plasma levels of LIF or Gal3 were not significantly affected by spleen sympathectomy (Supplementary information, Fig. S8c). On the other hand, this local removal of sympathetic inputs was sufficient to reduce PMN-MDSCs and M-MDSCs in the spleens (Fig. 6b) and also in the blood circulation (Fig. 6c) of tumor-bearing mice, suggesting that sympathetic signaling in the spleen facilitated the generation of MDSCs.

Finally, we FACS-sorted PMN-MDSCs and M-MDSCs from the spleens of mouse allograft models for in vitro cultures. Interestingly, the sympathetic neurotransmitter norepinephrine (NE) boosted the expression of immunosuppressive genes in MDSCs, e.g., *iNOS2*, *Arg1*, and *Il-10*. This neuroimmune effect could be entirely abrogated by the β2-adrenergic receptor antagonist propranolol (Fig. 6d). Meanwhile, we found that the plasma LIF or Gal3 levels of tumor-bearing mice were not affected by the exogenous NE treatment (Supplementary information, Fig. S8c), confirming that the release of LIF or Gal3 by cancer cells was independent of sympathetic signaling. These results elucidated that sympathetic signaling could directly promote the immunosuppressive function of MDSCs.

## Discussion

It has become recognized that despite the diverse mutational status of cancer cells, those that abundantly express neoantigens can effectively elicit the body’s antitumor immunity, which leads to tumor suppression and elimination.^33–37^ Indeed, therapeutic strategies aiming at the common pathways of antitumor immunity, e.g., PD-L1/PD1 or CTLA-4 signaling, have led to effective treatments or even a cure in the past decades.^35,38,39^ Meanwhile, evidence has begun to demonstrate that the nervous system may significantly influence the prognosis of many cancer types, and such cancer–neural interactions are emerging as a frontier of biomedical research.^1–3^

Previous studies in the field have documented diverse mechanisms of cancer cells engaging neural signals. However, recent studies implicated that different cancer types could share specific signaling events to communicate with the nervous system.^16–18^ Therefore, in contrast to the “uniqueness” of each cancer type, our current study reported an overlapping pattern of brain responses in multiple mouse models of peripheral tumors. Moreover, through the multi-omic screening, we successfully identified LIF and Gal3 as the key cytokines released by those cancer cells that robustly trigger neural activities in specific brain regions. Of importance, significant upregulation of the plasma levels of these two cytokines could be similarly observed in patients with different cancers. Moreover, we showed that pharmacologic or genetic blockage of cancer cell-derived LIF or Gal3 signaling to the brain would strongly inhibit tumor progression. These findings have suggested targeting the specific events of cancer–neural crosstalk for diagnostic or therapeutic benefits, i.e., cancer neurotherapy, conceptually analogous to the broad implementation of cancer immunotherapies.

We note that the detailed mechanisms by which LIF and Gal3 induce brain responses warrant more in-depth investigations. Notably, in the multi-omic screening of cancer cell-derived signaling molecules, we analyzed the expression of the putative receptor(s) for each candidate in the published scRNA-seq dataset of mouse trigeminal ganglionic neurons,^25^ whose axonal projections control Me5-L/-R and 5N-L/-R, the signature regions activated by multiple peripheral tumors. This scRNA-seq analysis revealed that the LIF receptor (*Lifr*) is highly expressed in those neuronal populations (Fig. 1f). Further, the published in situ hybridization data reveals the *Lifr* expression in the PVN neurons (Supplementary information, Fig. S8d), a central brain region of eliciting efferent sympathetic signals. These observations together imply that LIF may act directly via its receptor to induce brain activation. Future research exploiting mouse models with the neuron-specific deletion of *Lifr* will help delineate how LIF mediates brain responses to peripheral cancers. On the other hand, the precise identity of Gal3 receptor(s) is currently under debate,^40–42^ rendering it a challenge to elucidate the molecular mechanism of Gal3 action on the brain. It is possible that Gal3 can function via its putative receptor(s) expressed by neurons. At the same time, Gal3 may act as a “carrier” to facilitate the transport of LIF from the blood circulation across the blood-brain barrier into the brain. Such intriguing possibilities await future studies on the characterization of Gal3 receptor(s) in the context of cancer–neural crosstalk.

In sum, this study has elucidated a novel, shared mechanism of multiple cancer types hijacking neural signals to promote tumor progression (Fig. 6e), which has broad implications for our better understanding of the complexity of cancer neuroscience.

## Materials and methods

### Human blood samples

Blood samples from cancer patients or healthy donors were collected in compliance with the protocols approved by the Institutional Ethics Committees of Peking University Third Hospital or Peking University People’s Hospital. Informed consent was obtained from all the involved participants.

### Mouse information and procedures

All the experimental procedures in mice were performed in compliance with the protocols approved by the Institutional Animal Care and Use Committee of Peking University. Mice were maintained on the 12 h/12 h light/dark cycle (light period 7:00 a.m.–7:00 p.m.), with the standard chow diet and water available ad libitum. Mice utilized in experiments were 8–12 weeks old unless otherwise specified. C57BL/6, BALB/c, and FVB wild-type mice were purchased from the Charles River International. *Th-Cre;TrkA*^*fl/fl*^ and control *Th-Cre;TrkA*^*+/+*^ littermates were generated as previously reported.^31,32^ *MMTV-PyMT*^*+/−*^ male mice on the FVB background were obtained from the Jackson Laboratory (#002374) and in-house bred with FVB wild-type female mice to produce *MMTV-PyMT*^*+/–*^ female mice. *MMTV-PyMT*^*+/−*^ female mice of 5 weeks old were examined every other day for palpable mammary lumps, and the mice were euthanized for tissue analyses at 8 weeks after tumor onset.

For the procedure of spleen sympathectomy, C57BL/6 wild-type mice were anesthetized with 3% isoflurane, and the abdominal skin was shaved and prepared with iodine and alcohol. A skin incision was made below the left 12th-rib level, followed by another incision on the peritoneum to expose the spleen. Sympathetic branches entering the spleen were crushed with a pair of fine forceps (Fine Science Tools). The incisions on the peritoneum and the skin were then sutured. The sham surgery included all the steps except that sympathetic branches were untouched. The mice were utilized for experiments 10 days post sympathectomy.

For the administration of recombinant mouse LIF (Thermo Fisher Scientific) or Gal3 (BioLegend), each mouse was intraperitoneally injected with the indicated amounts of the proteins in 100 μL sterile saline.

For the treatment of the LIF inhibitor EC330 (MedChemExpress) or the Gal3 inhibitor GB1107 (MedChemExpress), tumor-bearing mice were daily administered with EC330 at 1 mg/kg of body weight or GB1107 at 5 mg/kg of body weight via intraperitoneal injection.

For the treatment of sympathetic neurotransmitter NE (Sigma), tumor-bearing mice were administered with NE at 1 mg/kg of body weight via intraperitoneal injection. Plasma levels of LIF or Gal3 were measured 4 h after the NE treatment.

### Cancer cell lines and allograft models

LLC cells, RM1 prostate cancer cells, MC38 colon cancer cells, and 4T1 breast cancer cells were purchased from the Chinese National Infrastructure of Cell Line Resource and tested negative for mycoplasma. LLC, RM1, MC38, and 4T1 cells were cultured in Dulbecco’s Modified Eagle Medium (DMEM; Gibco) supplemented with 10% heat-inactivated fetal bovine serum (HI-FBS; Sigma), 100 U/mL penicillin, and 100 μg/mL streptomycin.

For the genetic deletion of LIF or Gal3, each cancer cell line was transiently transfected with pSpCas9(BB)-2A-GFP (PX458; Addgene) expressing *Lif* sgRNA (GCATGGGTGGCGTATGGCAC) or *Gal3* sgRNA (TCAAGGATATCCGGGTGCAT). GFP-positive cells were sorted on the BD FACSAria at 16 h post transfection for single-cell clonal selection. Genetic deletion of *Lif* or *Gal3* allele was verified by DNA sequencing. In vitro cultured LIF KO or Gal3 KO cell lines exhibited the proliferation rates comparable to their parental “wild-type” cell lines as determined by the Cell Counting Kit-8 (Beyotime).

For the heterotopic allograft models of LLC, RM1, or MC38 cells, 5 × 10^5^ cancer cells in 50 μL DMEM were mixed with 50 μL Matrigel (Corning) and injected below the right forelimb of each C57BL/6 male mouse. For the heterotopic allograft model of 4T1 cells, 5 × 10^5^ cancer cells in 50 μL DMEM were mixed with 50 μL Matrigel and injected below the right forelimb of each BALB/c female mouse. For the orthotopic allograft model of LLC cells, 2.5 × 10^5^ cancer cells in 25 μL DMEM were mixed with 25 μL Matrigel and then intrathoracically injected into the left lobe of the lung of each C57BL/6 male mouse following the reported method.^22^ For the orthotopic allograft model of RM1 cells, 1 × 10^5^ cancer cells in 10 μL DMEM were mixed with 10 μL Matrigel and inoculated into the anterior lobes of the prostate of each C57BL/6 male mouse according to the reported procedure.^23^ Tumor dimensions of the heterotopic allograft models of LLC, RM1, MC38, or 4T1 cells were measured every 3–5 days, and tumor volumes were calculated as width (mm) × width (mm) × length (mm)/2.

For the implantation of a pseudo-tumor, ~1000 mm^3^ medical-grade silicone was subcutaneously implanted below the right forelimb of each C57BL/6 male mouse.

### RNA-seq analyses

Total RNAs of the tumors of mouse LLC, RM1, MC38, or 4T1 allograft models or *MMTV-PyMT*^*+/−*^ mice were extracted by the RNeasy Mini Kit (Qiagen). All the RNA samples were then subjected to single-end RNA-seq by the BGI Genomics. The RNA-seq data have been deposited to the Sequence Read Archive (https://www.ncbi.nlm.nih.gov/sra) with the accession number PRJNA990369 and PRJNA1080296.

### Proteomic analyses

LLC, RM1, MC38, or 4T1 cells were cultured in DMEM supplemented with 1% insulin-transferrin-selenium (Thermo Fisher Scientific), 100 U/mL penicillin, and 100 μg/mL streptomycin for 24 h. The culture media were harvested and 0.22-μm filtered before the acetone precipitation of total proteins. Protein precipitates were then subjected to trypsin digestion and proteomic analyses by the Orbitrap Exploris 480.

### ELISA

Human or mouse blood samples were collected in lithium heparin tubes and immediately centrifuged at 4000× *g* for 5 min. Plasma samples were then measured by mouse LIF ELISA kit (#MM-0158M2, MEIMIAN), mouse Galectin-3 ELISA kit (#MM-0808M2, MEIMIAN), human LIF ELISA kit (#MM-0083H2, MEIMIAN), or human Galectin-3 ELISA kit (#MM-13408H2, MEIMIAN) according to the manufacturer’s instructions.

### Immunostaining of mouse brain tissues

Brain tissues were acutely dissected from the mice under the indicated conditions and immediately fixed in phosphate-buffered saline (PBS) containing 3.7% paraformaldehyde at room temperature for 4 h. The tissues were cryopreserved in PBS containing 30% sucrose at 4 °C overnight before 16-μm sectioning. Brain sections were then immunostained with rabbit anti-p-S6 (#4858, Cell Signaling Technology), followed by Alexa Fluor 488-conjugated donkey anti-rabbit IgG secondary antibody (#A32790, Thermo Fisher Scientific). Immunostained brain sections were scanned by the Axio Scan Z1. p-S6 immunostaining in the indicated brain regions was quantified by ImageJ (https://imagej.net/ij) and normalized to the control condition of each experiment.

### FACS

PMN-MDSCs (CD45^+^ CD11b^+^ Ly6G^+^ Ly6C^−^), M-MDSCs (CD45^+^ CD11b^+^ Ly6C^+^ Ly6G^−^), and CD8^+^ T cells (CD45^+^ CD3^+^ CD8^+^ CD4^−^ NK1.1^−^) in the mouse blood, spleens, or tumors under the indicated conditions were stained by the FACS antibodies and processed on the BD LSRFortessa. The FACS data were analyzed by FlowJo (https://www.flowjo.com). FACS antibodies utilized in the experiments were CD45-PE (#103106, BioLegend), CD45-APC-Cy7 (#103116, BioLegend), CD11b-FITC (#101206, BioLegend), Ly6G-APC (#17-9668-82, eBioscience), Ly6C-APC-Cy7 (#128026, BioLegend), CD3-PE (#12-0032-82, eBioscience), CD4-APC (#100412, BioLegend), and CD8-FITC (#100706, BioLegend).

### In vitro treatments

PMN-MDSCs and M-MDSCs in the mouse spleens under the indicated conditions were FACS-sorted on the BD FACSAria. The cells were in vitro cultured in RPMI-1640 (Gibco) supplemented with 10% HI-FBS, 100 U/mL penicillin and 100 μg/mL streptomycin, and then treated with 10 μM NE or 20 μM propranolol (Selleck Chemicals) for 1 h. Total RNAs of the cells were extracted by the RNeasy Mini Kit and analyzed by the SYBR Green Real-Time PCR Kit (Thermo Fisher Scientific). *Gapdh* mRNA levels were utilized as the internal control.

### Statistical methods

Student’s *t*-test (two-tailed unpaired) or ANOVA with post hoc test was performed using GraphPad Prism 9.5.0 (http://www.graphpad.com/scientific-software/prism). All the data points represent biological replicates. Statistical details of the experiments are included in figure legends.

### Supplementary information

Supplementary information, Figure S1

Supplementary information, Figure S2

Supplementary information, Figure S3

Supplementary information, Figure S4

Supplementary information, Figure S5

Supplementary information, Figure S6

Supplementary information, Figure S7

Supplementary information, Figure S8

Supplementary information, Table S1
