# Supplementary material for: Multiple cancer cell types release LIF and Gal3 to hijack neural signals
Source: Cell Res. 2024 Mar 11;34(5):345–54. doi: 10.1038/s41422-024-00946-z (PMC11061112; doi:10.1038/s41422-024-00946-z)
Supplement: Supplementary file 1 — Supplementary information, Figure S1 [file 41422_2024_946_MOESM1_ESM.pdf]

Figure S1

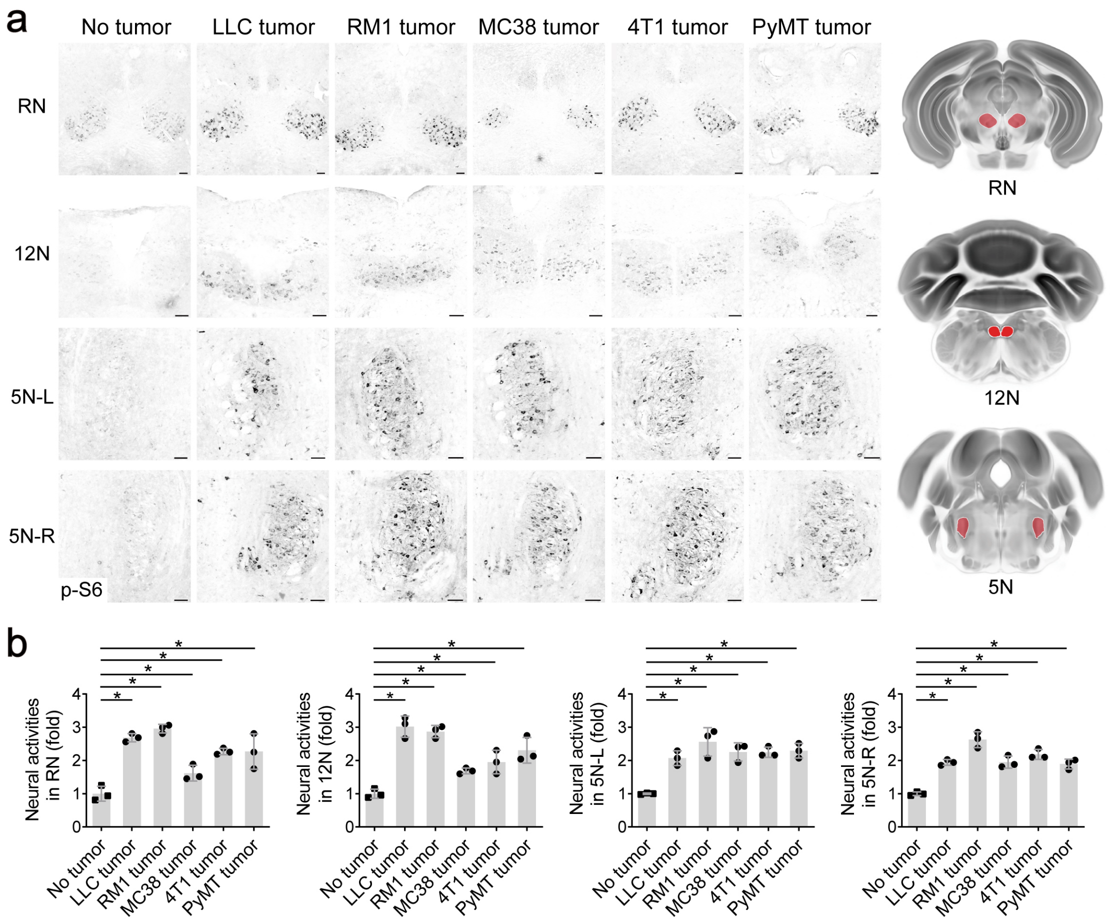

Supplementary information, Figure S1 An overlapping pattern of brain responses in mouse models of peripheral cancers.

Mouse allograft models of LLC, RM1, MC38, or 4T1 cells and the *MMTV-PyMT* mouse model were utilized. Brain responses were assessed by the p-S6 immunostaining. a Representative images of the RN, 12N, and 5N-L/-R were shown **(a)**. Scale bars, 100μm. Neural activities in the indicated brain regions were quantified **(b)**. mean ± SD, one-way ANOVA test, * $p < 0.05$.
