# Supplementary material for: Multiple cancer cell types release LIF and Gal3 to hijack neural signals
Source: Cell Res. 2024 Mar 11;34(5):345–54. doi: 10.1038/s41422-024-00946-z (PMC11061112; doi:10.1038/s41422-024-00946-z)
Supplement: Supplementary file 2 — Supplementary information, Figure S2 [file 41422_2024_946_MOESM2_ESM.pdf]

Figure S2

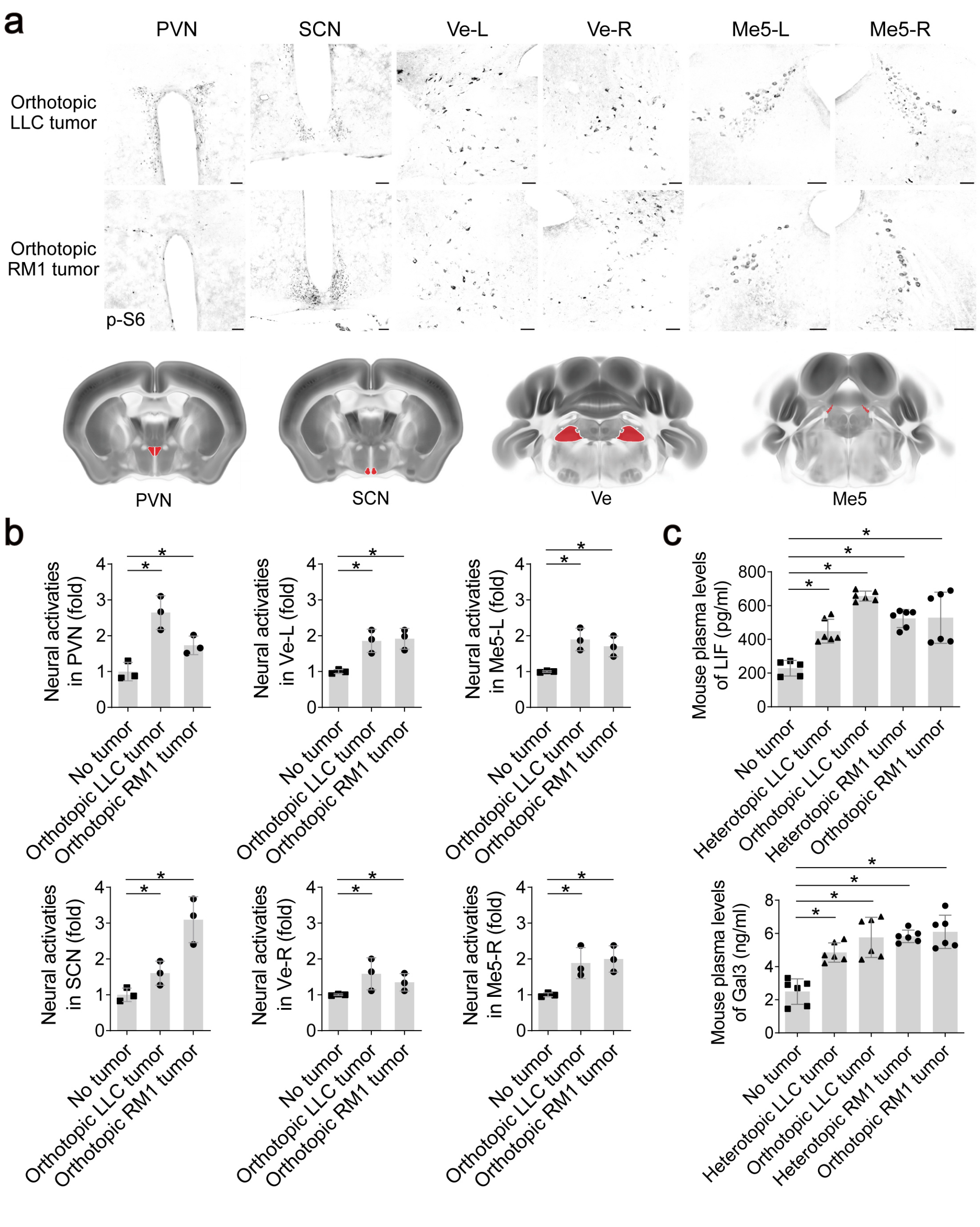

Supplementary information, Figure S2 Brain responses in the mouse models of orthotopic allograft tumors.

a, b C57BL/6 wild-type mice were utilized for LLC or RM1 orthotopic allograft models.

Brain responses were assessed by the p-S6 immunostaining. Representative images of the PVN, SCN, Ve-L/-R, and Me5-L/-R were shown **(a)**. Scale bars, 100μm. Neural activities in the indicated brain regions were quantified **(b)**. mean ± SD, one-way ANOVA test, * $p < 0.05$. **c** C57BL/6 wild-type mice were utilized for the heterotopic or orthotopic allograft models of LLC or RM1 cells. Plasma levels of LIF and Gal3 in the mice of indicated conditions were examined by ELISA. mean ± SD, one-way ANOVA test, * $p < 0.05$.
