# Supplementary material for: Multiple cancer cell types release LIF and Gal3 to hijack neural signals
Source: Cell Res. 2024 Mar 11;34(5):345–54. doi: 10.1038/s41422-024-00946-z (PMC11061112; doi:10.1038/s41422-024-00946-z)
Supplement: Supplementary file 3 — Supplementary information, Figure S3 [file 41422_2024_946_MOESM3_ESM.pdf]

Figure S3

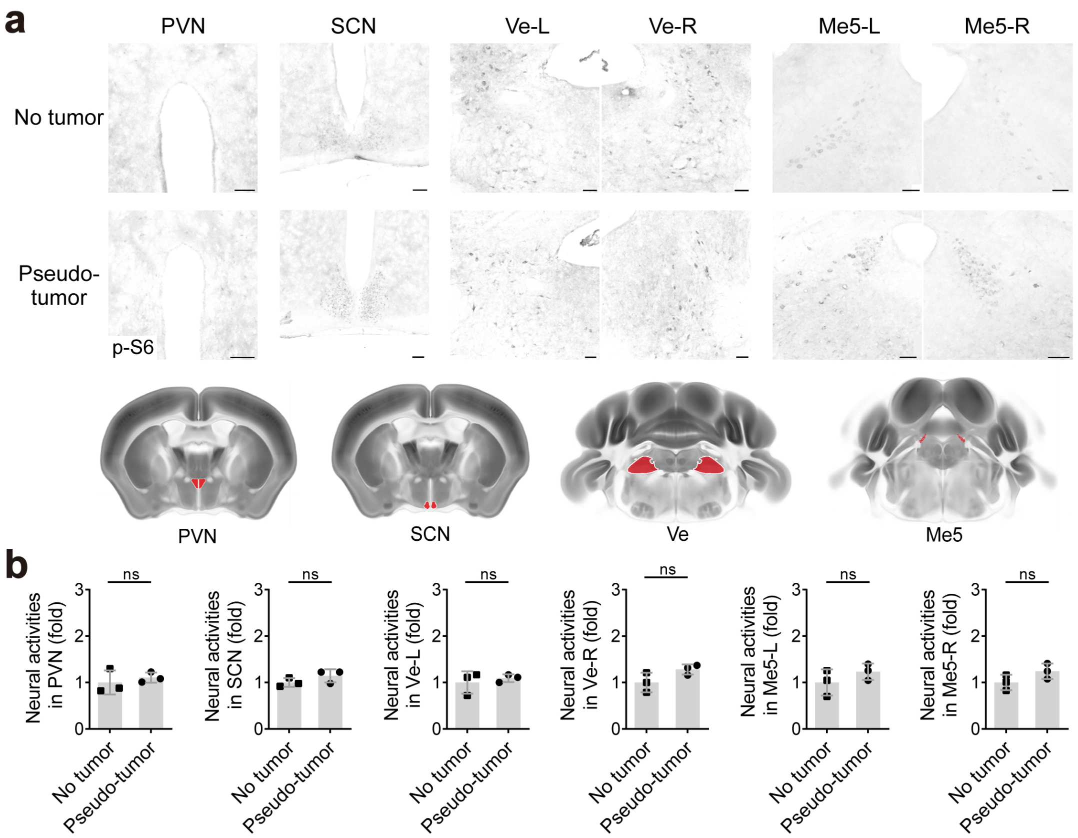

Supplementary information, Figure S3 A pseudo-tumor fails to trigger brain responses.

a, b C57BL/6 wild-type mice were subcutaneously implanted with medical-grade silicone to mimic a peripheral tumor. Brain responses were assessed by the p-S6 immunostaining. Representative images of the PVN, SCN, Ve-L/-R, and Me5-L/-R were shown **(a)**. Scale bars, 100μm. Neural activities in the indicated brain regions were quantified **(b)**. mean ± SD, Student's *t*-test, ns not significant.
