# Supplementary material for: Multiple cancer cell types release LIF and Gal3 to hijack neural signals
Source: Cell Res. 2024 Mar 11;34(5):345–54. doi: 10.1038/s41422-024-00946-z (PMC11061112; doi:10.1038/s41422-024-00946-z)
Supplement: Supplementary file 4 — Supplementary information, Figure S4 [file 41422_2024_946_MOESM4_ESM.pdf]

Figure S4

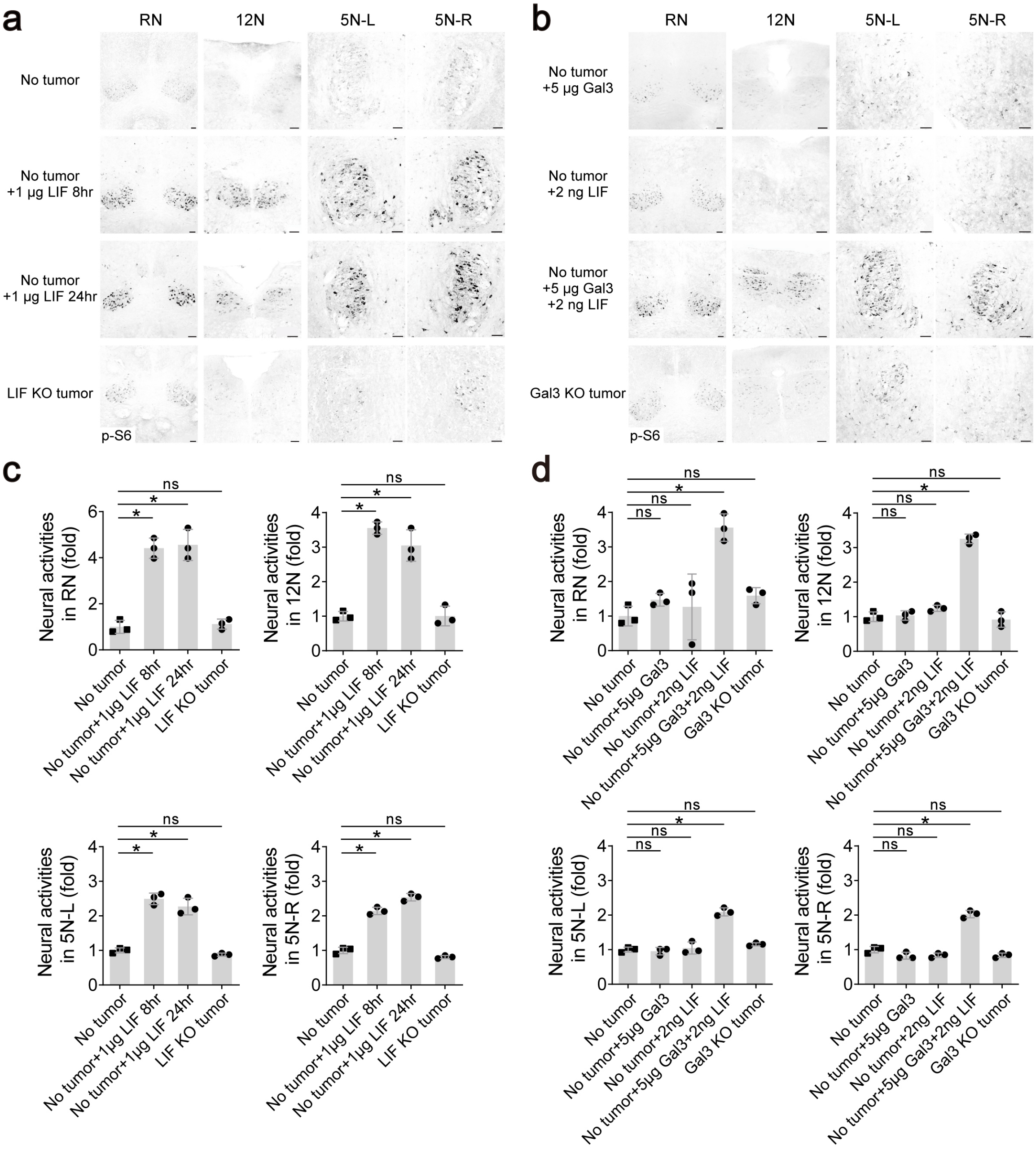

Supplementary information, Figure S4 LIF and Gal3 are the cancer cell-derived factors that cooperatively activate specific brain regions.

a-d Non-tumor-bearing C57BL/6 wild-type mice were administered with the indicated amounts of recombinant mouse LIF or Gal3 proteins. In comparison, mouse allograft

models of LLC cancer cells with the genetic deletion of LIF or Gal3 were utilized. Brain responses were assessed by the p-S6 immunostaining. Representative images of the RN, 12N, and 5N-L/-R were shown **(a, b)**. Scale bars, 100μm. Neural activities in the indicated brain regions were quantified **(c, d)**. mean ± SD, one-way ANOVA test, ns not significant, * $p < 0.05$.
