# Supplementary material for: Multiple cancer cell types release LIF and Gal3 to hijack neural signals
Source: Cell Res. 2024 Mar 11;34(5):345–54. doi: 10.1038/s41422-024-00946-z (PMC11061112; doi:10.1038/s41422-024-00946-z)
Supplement: Supplementary file 5 — Supplementary information, Figure S5 [file 41422_2024_946_MOESM5_ESM.pdf]

Figure S5

```
LLC-LIF KO-Line 1
          gcagggagccctcttcccatcacccctgtaaatgccacctgtgccatacgccacccatgccacggcaacctcatgaaccagatcaagaatcaactggcac
allele 1  gcagggagccctcttcccatcacccctgtaaatgccacctgtgccatacgccaccca-----cggcaacctcatgaaccagatcaagaatcaactggcac   [-5bp]
allele 2  gcagggagccctcttcccatcacccctgtaaatg-----------------------------------------aaccagatcaagaatcaactggcac   [-41bp]

LLC-LIF KO-Line 2
          gcagggagccctcttcccatcacccctgtaaatgcacctgtgccatacgccacccatgccacggcaacctcatgaaccagatcaagaatcaactggcacagctcaatggcagcgccaatgctctcttcatttcctatgtaagttactttcctggggta
allele 1  gcagggagccctcttcccatcacccctgtaaatgcacctg------------------------------------------------------------------------------------------------------------------gggta   [-113bp]
allele 2  gcagggagccctcttcccatcacccctgtaaatgcacctgtg-------------------------------- aaccagatcaagaatcaactggcacagctcaatggcagcgccaatgctctcttcatttcctatgtaagttactttcctggggta   [-32bp]

RM1-LIF KO-Line 1
          gcagggagccctcttcccatcacccctgtaaatgccacctgtgccatacgccacccatgccacggcaacctcatgaaccagatcaagaatcaactggcacagctcaatggcagcgc
allele 1  gcagggagccctcttcccatcacccctgtaaatgccacctgt-ccatacgccacccatgc-acggcaacctcatgaaccagatcaagaatcaactggcacagctcaatggcagcgc   [-1bp] [-1bp]
allele 2  gcagggagccctcttcccatcacccctgtaaatgccacctgtgccatacgccaccc-------ggcaacctcatgaaccagatcaagaatcaactggcacagctcaatggcagcgc   [-7bp]

RM1-LIF KO-Line 2
          gcagggagccctcttccc  atcacccctgtaaatgccacctgtgcca      tacgccacccatgccacggcaacctcatgaaccagatcaagaatcaactggcacagctcaatggcagcgc
allele 1  gcagggagccctcttcccg atcacccctgtaaatgccacctgtgccaccca-------------ccacggcaacctcatgaaccagatcaagaatcaactggcacagctcaatggcagcgc   [+1bp] [+4bp] [-13bp]
allele 2  gcagggagccctcttccc  atcacccctgtaaatgccacctgt-cca      tacgccacccatgc-acggcaacctcatgaaccagatcaagaatcaactggcacagctcaatggcagcgc   [-1bp] [-1bp]

LLC-Gal3 KO-Line 1
          aacccaaaccctcaaggatatccgggtgc                                                                                                                       atggggg
allele 1  aacccaaaccctcaaggatatccgggtgcgcgtaaccgctacacttgccagcgccttagcgcccgctcctttcgctttcttcccttcctttctcgccacgttcgccggctttccccgtcaagctctaaatcgggggctccctttagggttccgatttagtgctttac---ggcgg   [+138bp] [-2bp]
allele 2  aacccaaaccctcaaggatatccgggtg-                                                                                                                        atggggg   [-1bp]

LLC-Gal3 KO-Line 2
          aacccaaaccctcaaggatatccgggtgc  atgggggaaccagcctggggcagggggctacccaggggctgcttatcctgg
allele 1  aacccaaaccctcaaggatatccgggtgcc atgggggaaccagcctggggcagggggctacccaggggctgcttatcctgg   [+1bp]
allele 2  aacccaaaccctcaaggata--------c  atgggggaaccagcctggggcagggggctacccaggggctgcttatcctgg   [-8bp]

RM1-Gal3 KO-Line 1
          aacccaaaccctcaaggatatccgggtgcatgggggaaccagcctggggcagggggctacccaggggctgcttatcctgg
allele 1  aacccaaaccctca----------------tgggggaaccagcctggggcagggggctacccaggggctgcttatcctgg   [-16bp]
allele 2  aacccaaaccctcaaggatatccgggtg--tgggggaaccagcctggggcagggggctacccaggggctgcttatcctgg   [-2bp]

RM1-Gal3 KO-Line 2
          aacccaaaccctcaaggatatccgggtgcatgggggaaccagcctggggcagggggctacccaggggctgcttatcctgg
allele 1  aacccaaaccctcaaggatatccgggt--catgggggaaccagcctggggcagggggctacccaggggctgcttatcctgg   [-1bp]
allele 2  aacccaaaccctcaaggatatccggg----------------------gcagggggctacccaggggctgcttatcctgg   [-22bp]

MC38-LIF KO-Line 1
          aaacacggggcagggagccctcttcccatcacccctgtaaatgccacctgtgccatacgccacccatgccacggcaacctcatga
allele 1  aaacacggggcagggagccctcttcccatcacccctgtaaatgccacctgtgc- atacgccacccat- ccacggcaacctcatga   [-1bp] [-1bp]
allele 2  aaacacggg- cagggagccctcttcccatcacccctgtaaatgccacctgtgccatacgccacc---------------tcatga   [-1bp] [-15bp]

MC38-LIF KO-Line 2
          cccctgtaaatgccacctgtgcc  atacgccacccatgcc  acggcaacctcatgaaccagatcaagaat
allele 1  cccctgtaaatgccacct----------------------------------gaaccagatcaagaat   [-34bp]
allele 2  cccctgtaaatgccacctgtgcccatacgccacccatgcccacggcaacctcatgaaccagatcaagaat   [+1bp] [+1bp]

4T1-LIF KO-Line 1
          ggggcagggagccctcttcccatcacccctgtaaatgccacctgtgccatacgccacccatgccacggcaacctcatgaaccagatcaagaat
allele 1  ggggcagggagccctcttcccatcacccctgtaaatgccacct -------------------------------catgaaccagatcaagaat   [-31bp]
allele 2  ggggcagggagccctcttcc --------------------------------------------------------------agatcaagaat   [-62bp]

4T1-LIF KO-Line 2
          ggggcagggagccctcttcccatcacccctgtaaatgccacctgtgccatacgccacccatgccacggcaacctcatgaaccagatcaagaat
allele 1  ggggcagggagccctcttcccatcacccctgtaaatgcca --------- tacgccacccat- ccacggcaacctcatgaaccagatcaagaat   [-9bp] [-1bp]
allele 2  ggggcagggagccctcttcccatcacccctgtaaatgccacctgtg ---- acgccacccat- ccacggcaacctcatgaaccagatcaagaat   [-4bp] [-1bp]
```

Supplementary information, Figure S5 Genetic deletion of LIF or Gal3 in mouse cancer cell lines.

DNA sequencing results of indicated cancer cell lines were shown.
