# Supplementary material for: Multiple cancer cell types release LIF and Gal3 to hijack neural signals
Source: Cell Res. 2024 Mar 11;34(5):345–54. doi: 10.1038/s41422-024-00946-z (PMC11061112; doi:10.1038/s41422-024-00946-z)
Supplement: Supplementary file 6 — Supplementary information, Figure S6 [file 41422_2024_946_MOESM6_ESM.pdf]

Figure S6

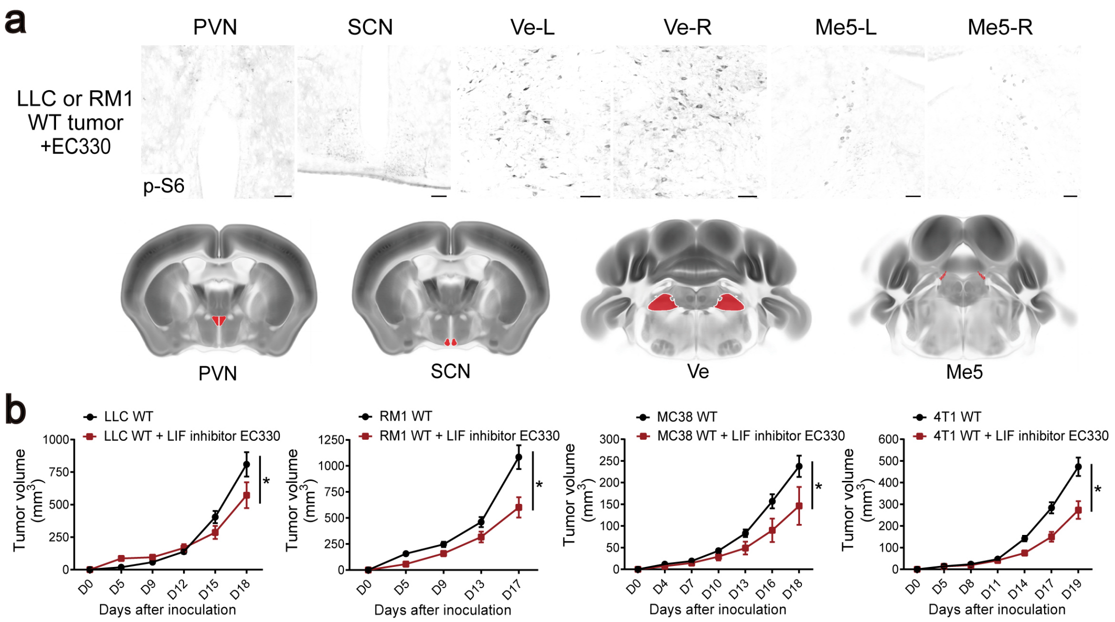

Supplementary information, Figure S6 Pharmacologic blockage of the LIF signal inhibits tumor progression.

a C57BL/6 wild-type mice in LLC or RM1 allograft models were treated with the LIF inhibitor EC330, and brain responses were assessed by the p-S6 immunostaining. Representative images of the PVN, SCN, Ve-L/-R, and Me5-L/-R were shown. Scale bars, 100μm. **b** Mouse allograft models of LLC, RM1, MC38, or 4T1 cells were treated with the LIF inhibitor EC330. Tumor growth rates of the indicated conditions were monitored. n = 10, mean ± SD, two-way ANOVA test, * $p < 0.05$.
