# Supplementary material for: Multiple cancer cell types release LIF and Gal3 to hijack neural signals
Source: Cell Res. 2024 Mar 11;34(5):345–54. doi: 10.1038/s41422-024-00946-z (PMC11061112; doi:10.1038/s41422-024-00946-z)
Supplement: Supplementary file 7 — Supplementary information, Figure S7 [file 41422_2024_946_MOESM7_ESM.pdf]

Figure S7

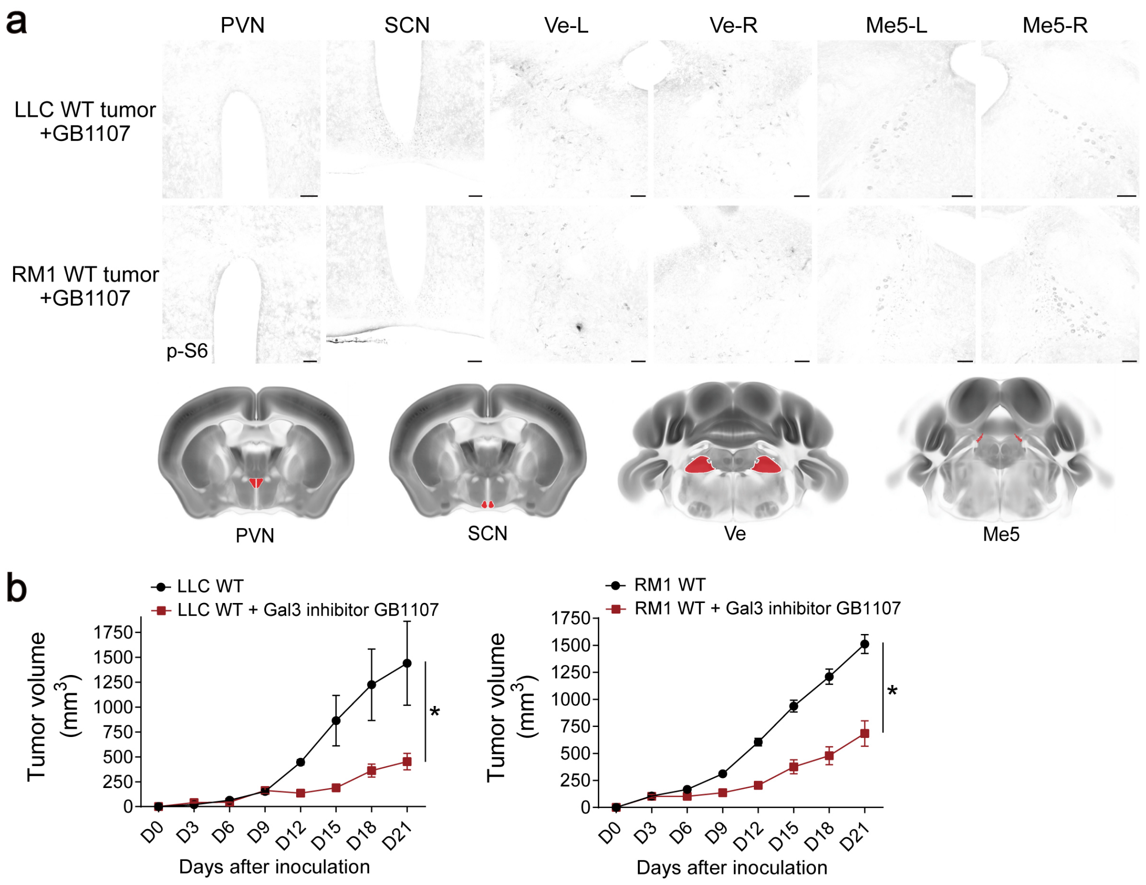

Supplementary information, Figure S7 Pharmacologic blockage of the Gal3 signal delays tumor progression.

a, b C57BL/6 wild-type mice in LLC or RM1 allograft models were treated with the Gal3 inhibitor GB1107. Brain responses were assessed by the p-S6 immunostaining, and representative images of the PVN, SCN, Ve-L/-R, and Me5-L/-R were shown **(a)**. Scale bars, 100μm. Tumor growth rates of the indicated conditions were monitored **(b)**. n = 10, mean ± SD, two-way ANOVA test, * $p < 0.05$.
