# Supplementary material for: Multiple cancer cell types release LIF and Gal3 to hijack neural signals
Source: Cell Res. 2024 Mar 11;34(5):345–54. doi: 10.1038/s41422-024-00946-z (PMC11061112; doi:10.1038/s41422-024-00946-z)
Supplement: Supplementary file 8 — Supplementary information, Figure S8 [file 41422_2024_946_MOESM8_ESM.pdf]

Figure S8

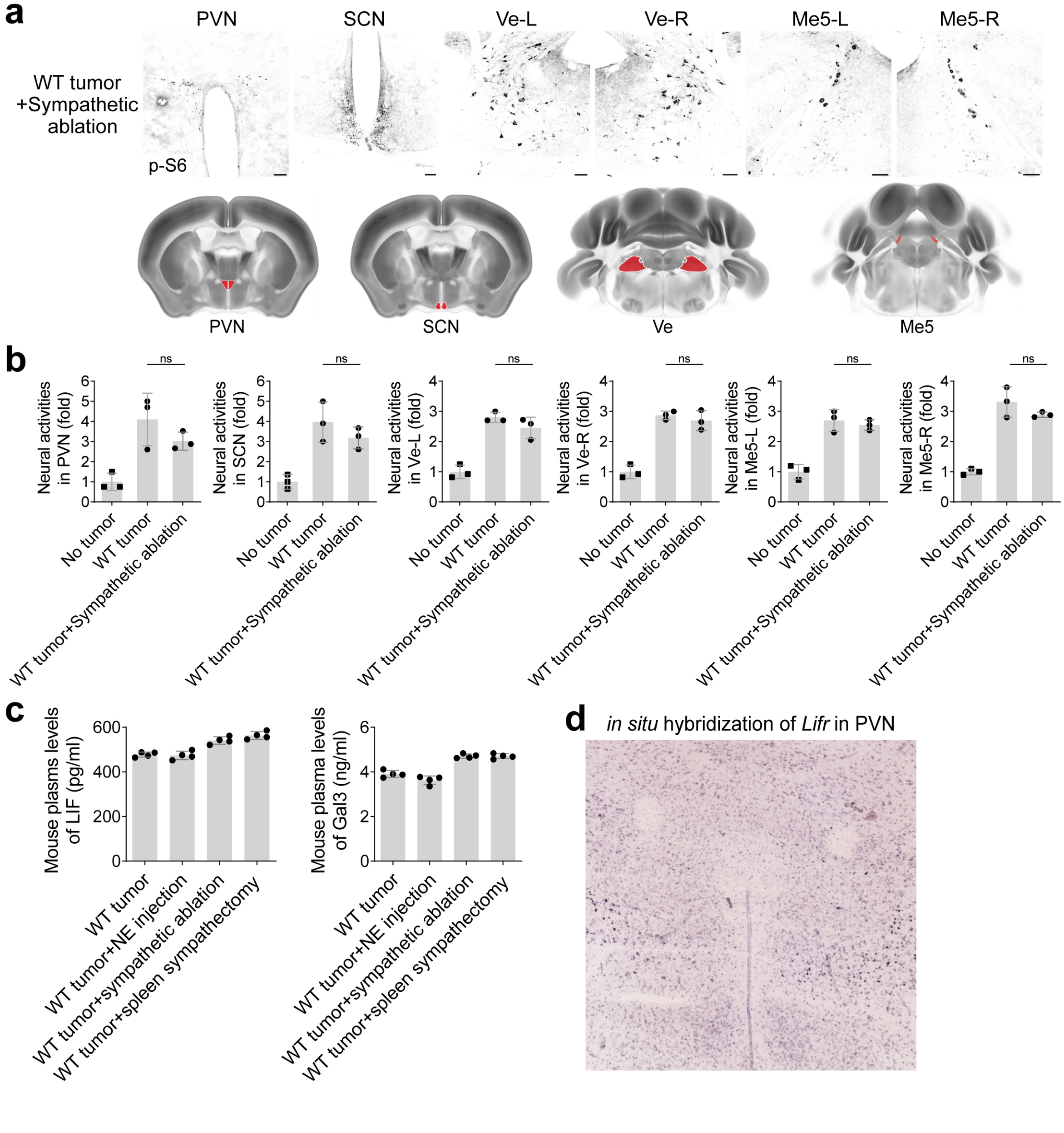

Supplementary information, Figure S8 Cancer cell-derived LIF and Gal3 act via downstream sympathetic signals.

a, b *Th-Cre; TrkA*$^{fl/fl}$ (sympathetic ablation) and control *Th-Cre; TrkA*$^{+/+}$ littermates were examined in LLC allograft models, and brain responses were assessed by the p-S6

immunostaining. Representative images of the PVN, SCN, Ve-L/-R, and Me5-L/-R of tumor-bearing mice were shown **(a)**. Scale bars, 100μm. Neural activities in the indicated brain regions were quantified **(b)**. mean ± SD, one-way ANOVA test, ns not significant. **c** LLC allograft models were established in C57BL/6 wild-type mice, *Th-Cre; TrkA$^{fl/fl}$* mice (sympathetic ablation), or C57BL/6 mice with spleen sympathectomy. In addition, the tumor-bearing C57BL/6 wild-type mice were treated with the sympathetic neurotransmitter norepinephrine (NE injection). Plasma levels of LIF and Gal3 in the mice of indicated conditions were compared by ELISA. mean ± SD. **(d)** *Lifr* expression in the mouse PVN region, as reported by the *in situ* hybridization (Allen Institute for Brain Science).
