# Supplementary material for: Multiple cancer cell types release LIF and Gal3 to hijack neural signals
Source: Cell Res. 2024 Mar 11;34(5):345–54. doi: 10.1038/s41422-024-00946-z (PMC11061112; doi:10.1038/s41422-024-00946-z)
Supplement: Supplementary file 9 — Supplementary information, Table S1 [file 41422_2024_946_MOESM9_ESM.pdf]

Table S1. Candidate factors identified by the multi-omic screen.

Protein number	Protein name	Receptor	Receptor' protein number	Receptor' gene ID
Q05793	heparan sulfate proteoglycan core protein			
P01027	Complement C3	C3aR	O09047	12267
Q61147	Ceruloplasmin			
P11276	Fibronectin			
Q61398	Procollagen C-endopeptidase enhancer 1			
P97298	Pigment epithelium-derived factor	PNPLA2	Q8BJ56	66853
Q61703	Inter-alpha-trypsin inhibitor heavy chain H2			
Q02819	Nucleobindin-1			
P27773	Protein disulfide-isomerase A3			
Q9R0E2	Procollagen-lysine,2-oxoglutarate 5-dioxygenase 1			
Q07797	Galectin-3-binding protein			
P21460	Cystatin-C			
P13020	Gelsolin			
P08113	Endoplasmin	TLR4, TLR9	Q9QUK6, Q9EQU3	21898, 81897
P20029	78 kDa glucose-regulated protein			
P09056	Leukemia inhibitory factor	LIFR	P42703	16880
Q61207	Sulfated glycoprotein 1			
P07214	SPARC			
P35441	Thrombospondin-1	CD36, CD47	Q08857, Q61735	12491, 16423
P47879	Insulin-like growth factor-binding protein 4			
P19221	Prothrombin	PAR1	P30558	112304
P08121	Collagen alpha-1(III) chain			
P10605	Cathepsin B			
Q9EPL2	Calsyntenin-1			
P28798	Granulins			
Q61704	Inter-alpha-trypsin inhibitor heavy chain H3			
P01887	Beta-2-microglobulin			
Q8BND5	Sulfhydryl oxidase 1			
Q9R118	Serine protease HTRA1			
Q08879	Fibulin-1			
Q04857	Collagen alpha-1(VI) chain			
P32261	Antithrombin-III			
Q61508	Extracellular matrix protein 1			
Q62356	Follistatin-related protein 1			
P01899	H-2 class I histocompatibility antigen, D-B alpha chain			
P08122	Collagen alpha-2(IV) chain			
P02463	Collagen alpha-1(IV) chain			
Q8BHN3	Neutral alpha-glucosidase AB			
P16110	Galectin-3			
P12032	Metalloproteinase inhibitor 1			
P06797	Cathepsin L1			
P07141	Macrophage colony-stimulating factor 1	CSF1R	P09581	12978

Q03366	C-C motif chemokine 7	CCR7	P47774	12775
P49182	Heparin cofactor 2			
P47880	Insulin-like growth factor-binding protein 6			
P12850	Growth-regulated alpha protein	CXCR2	P35343	54199
Q9R1Q9	V-type proton ATPase subunit S1			
Q6GQT1	Alpha-2-macroglobulin-P			
Q9CYN9	Renin receptor			
Q9Z0K8	Pantetheinase			
Q00493	Carboxypeptidase E			
Q99JR5	Tubulointerstitial nephritis antigen-like			
P25785	Metalloproteinase inhibitor 2			
Q9WVJ9	EGF-containing fibulin-like extracellular matrix protein 2			
Q60963	Platelet-activating factor acetylhydrolase			
P10148	C-C motif chemokine 2	CCRL2	O35457	12765
P11087	Collagen alpha-1(I) chain			
Q00623	Apolipoprotein A-I			
Q8QZR4	Out at first protein homolog			
Q9WV54	Acid ceramidase			
P29699	Alpha-2-HS-glycoprotein			
